# *In vivo *and *in silico *determination of essential genes of *Campylobacter jejuni*

**DOI:** 10.1186/1471-2164-12-535

**Published:** 2011-11-01

**Authors:** Aline Metris, Mark Reuter, Duncan JH Gaskin, Jozsef Baranyi, Arnoud HM van Vliet

**Affiliations:** 1 Institute of Food Research, Norwich Research Park, Colney Lane, Norwich NR4 7UA, UK

## Abstract

**Background:**

In the United Kingdom, the thermophilic *Campylobacter *species *C. jejuni *and *C. coli *are the most frequent causes of food-borne gastroenteritis in humans. While campylobacteriosis is usually a relatively mild infection, it has a significant public health and economic impact, and possible complications include reactive arthritis and the autoimmune diseases Guillain-Barré syndrome. The rapid developments in "omics" technologies have resulted in the availability of diverse datasets allowing predictions of metabolism and physiology of pathogenic micro-organisms. When combined, these datasets may allow for the identification of potential weaknesses that can be used for development of new antimicrobials to reduce or eliminate *C. jejuni *and *C. coli *from the food chain.

**Results:**

A metabolic model of *C. jejuni *was constructed using the annotation of the NCTC 11168 genome sequence, a published model of the related bacterium *Helicobacter pylori*, and extensive literature mining. Using this model, we have used *in silico *Flux Balance Analysis (FBA) to determine key metabolic routes that are essential for generating energy and biomass, thus creating a list of genes potentially essential for growth under laboratory conditions. To complement this *in silico *approach, candidate essential genes have been determined using a whole genome transposon mutagenesis method. FBA and transposon mutagenesis (both this study and a published study) predict a similar number of essential genes (around 200). The analysis of the intersection between the three approaches highlights the shikimate pathway where genes are predicted to be essential by one or more method, and tend to be network hubs, based on a previously published *Campylobacter *protein-protein interaction network, and could therefore be targets for novel antimicrobial therapy.

**Conclusions:**

We have constructed the first curated metabolic model for the food-borne pathogen *Campylobacter jejuni *and have presented the resulting metabolic insights. We have shown that the combination of *in silico *and *in vivo *approaches could point to non-redundant, indispensable genes associated with the well characterised shikimate pathway, and also genes of unknown function specific to *C. jejuni*, which are all potential novel *Campylobacter *intervention targets.

## Background

The rise of antibiotic resistance in pathogenic bacteria is a growing concern in the developed world necessitating knowledge-led approaches to identify new interventions and prevention strategies [1]. One of the common sources of pathogenic bacteria is food, with the foodborne zoonotic pathogens *Salmonella, Escherichia coli *and *Campylobacter *being prime examples. Although it can be contended whether the use of antibiotics in the food industry contributes to antimicrobial resistance, it is clear that food-borne pathogens also increasingly acquire resistance to antimicrobial interventions. Multidrug resistance in *Salmonella *is well documented [2,3]. For *Listeria*, antibiotic resistance has also been reported for strains isolated from food [4]. In *Campylobacter*, resistance to ampicillin, erythromycin, tetracycline, and ciprofloxacin have all been reported [5-7].

In Europe, *Campylobacter *was the most frequent cause of food-borne illness in 2007, with over 200,000 laboratory-confirmed cases [8] although the total number of cases is thought to be approximately eightfold higher. Infection by *Campylobacter *is thought to be largely due to the consumption of contaminated poultry either through poor food preparation hygiene or under-cooking [9]. While the symptoms associated with *C. jejuni *infection (diarrhoea, vomiting, and stomach pains) often only last between 2 to 5 days, sequelae of *C. jejuni *infection include more serious autoimmune diseases like Guillain-Barré syndrome, Miller-Fisher syndrome [9], and reactive arthritis [10]. While human infection often does not require antibiotic intervention, the organism is endemic in poultry and farm animals, and it would be advantageous to have treatment options before entry in the food chain.

One approach for the identification of new antibiotic targets for a particular bacterial pathogen is to identify non-redundant cellular functions or metabolic pathways that are indispensible for growth and/or survival of that organism; for example, key metabolic enzymes or cell wall synthesis proteins. In the post-genomic era, genome analysis makes both bioinformatic predictions and targeted mutagenesis strategies feasible, due to the availability of large, curated datasets. However, genome annotation is often incomplete and incorrect, and metabolic redundancy (alternative pathways or catalytic activities) can confound such rational approaches. For instance, a comprehensive study in *Salmonella *of essential genes required during infection showed that many enzymes are not essential, partly due to metabolic redundancy [11]. An alternative (experimental, high-throughput) strategy is the use of random approaches such as transposon mutagenesis to identify essential genes that would be needed to infect an animal model or to grow and proliferate [12-14].

*In silico*, essential genes of well characterised micro-organisms, such as *E. coli*, are predicted with high accuracy by Flux Balance Analysis (FBA) of the metabolic network [15]. FBA consists of the computation of the fluxes going through the metabolic reactions when the cells are in a homeostatic state [15]. The reactions are linked to the genes that encode the corresponding enzymes with Boolean relationships, and, a gene is predicted to be essential if *in silico *deletion results in negligible biomass [15]. According to Feist *et al.*, [16], about 90% of essential genes of *E. coli *can be predicted in a given environment. For micro-organisms other than the established model systems, the accuracy is lower (60-80%) [17,18]. Nevertheless, it provides insights into cellular metabolism, which can be useful to identify potential new drug targets [19].

In this study we have constructed a genome scale metabolic model of the food-borne pathogen *Campylobacter jejuni *and discuss this organism's metabolism. Additionally, we have combined both *in silico *and *in vivo *approaches to make predictions about essential genes. A published FBA model of the closely related organism *Helicobacter pylori *[18] provided the basis for a *Campylobacter *FBA model. The essential genes predicted from FBA of the reconstructed genome scale model of *C. jejuni *were compared to new experimentally generated transposon mutagenesis data, and a recently published independent whole genome transposon mutagenesis study [20]. Whilst the overlap between the three methods is comparatively small, the combination of these methods has drawn focus to the shikimate pathway, a known target pathway for new antimicrobial interventions in other bacterial pathogens [21].

## Results

### Metabolic network reconstruction

In a first step, we have reconstructed the metabolic network of *C. jejuni *based on the annotation of the NCTC 11168 genome sequence [22,23], a published model of the related bacterium *H. pylori *[18] and extensive literature mining. The resulting network is made of 536 reactions in total accounting for 388 genes and 467 metabolites, that is, it is of similar size as the model of *H. pylori *(see Table 1 for the number of reactions in this model as compared with the *H. pylori *model and the Additional file 1: model for a table with the model itself). In this section we will discuss specific areas of *C. jejuni *metabolism as present in, or predicted by, our model.

**Table 1:** Number of reactions in the *C. jejuni *model (this paper) and in the *H. pylori *model [18].

Type of reaction	*C. jejuni *model	*H. pylori *model
Metabolic	410 (367)	399 (317)
Transport	64 (32)	77 (38)
Sink/demands	3	3
Exchange	59	74
Total	536	553

The number of reactions associated to genes is given in brackets.

#### Central metabolism

The reactions of the central metabolism have been mainly drawn from the literature since contrarily to *H. pylori*, *C. jejuni *is predicted to contain a complete TCA cycle with some enzymes characteristic for anaerobes [24]. *C. jejuni *does not metabolise glucose [25] and the genome annotation suggests that the Embden-Meyerhof pathway only functions in gluconeogenesis [24], so overall the space of solution of fluxes is different from *H. pylori*. The respiratory chain of *C. jejuni *is more complex than that of *H. pylori *[26], and *C. jejuni *can use sulphite as an electron donor [27].

The biosynthesis of folate results in the production of glycolaldehyde, which can cause cell damage by electrophilic attack of negatively-charged molecules [28]. In the *H. pylori *and *E. coli *models, glycolaldehyde is converted to glycolate by a glycolaldehyde dehydrogenase and glycolate is degraded further through glyoxylate metabolism [16,18]. Alternatively glycolate can diffuse out of the cell in the case of *E. coli *[16]. Since a glyoxylate oxidase (Cj1213c) has been annotated [23] and since glyoxylate has been shown to be degraded via a malate synthase in *H. pylori *[29], we assumed a malate synthase in our model despite the genome lacking the corresponding annotation as in *H. pylori *[30,31].

#### Amino acid metabolism

The reactions for the amino acid metabolism have been mainly drawn from the genome annotation. Contrarily to *H. pylori, C. jejuni *seems to have the capacity to synthesize all the amino acids and vitamins it requires. This was shown experimentally with BIOLOG phenotype microarrays, where respiration was detected on growth medium containing salts and a carbon source only [32]. From the genome sequence, complete pathways for the synthesis of isoleucine, leucine and valine, are present in *C. jejuni*, whereas these pathways are incomplete or absent in *H. pylori*. In addition to the amino acids required by *H. pylori*, a complete pathway to synthesize histidine was found in *C. jejuni *(*cj0317, cj1315c, cj1597-99, cj1600-01, cj1603-04*) while orthologs of these genes are absent in the *H. pylori *genome. The gene for the last step of methionine synthesis is predicted to be present in *C. jejuni *(*cj1201*), so no demand reaction was included in the model to artificially consume S-adenosylmethione. In common with *H. pylori*, only one gene of the methionine salvage pathway was found to be present in *C. jejuni *(*cj0117*). In the iIT341 GSM/GPR *H. pylori *model, the pathway was nevertheless included to ensure the recycling of 5-methylthioadenosine, a by-product of spermidine biosynthesis, to methionine, based on the assumption that the pathways vary from one micro-organism to another. However it has recently been suggested that the last steps of spermidine biosynthesis in *C. jejuni *differ from the pathway proposed for *H. pylori *[33] eliminating the necessity for recycling 5-methylthioadenosine, so the methionine salvage pathway was not included in this model.

#### Nucleotide metabolism

The reactions for the nucleotide metabolism have been almost exclusively derived from the genome annotation. The pathway for the synthesis of IMP is more similar to that of *E. coli*, rather than that of *H. pylori*. Only a few genes have been annotated at the level of nucleotide inter-conversions such as *cj0293 *which has been predicted to encode for a nucleotidase [23]. However most of the products of the reactions catalysed by this enzyme are not reutilised in the metabolic network, they are dead-ends. So either *cj0293 *is incorrectly annotated, or genes encoding enzymes to utilize the products of the nucleotidase have not been annotated.

#### Vitamin and cofactor metabolism

In the *H. pylori *model iIT341 GSM/GPR, it was assumed that pimelate diffuses into the cell and that the first step of the synthesis of biotin is catalysed by pimelyl-CoA synthetase although no locus was found for such a gene [18]. Having no better alternative, we kept the assumption of the *H. pylori *model. *C. jejuni *does not contain ubiquinone, but uses menaquinone 6 and a methyl-substituted menaquinone [34]. It has been shown that for *Streptomyces coelicolor, H. pylori, C. jejuni *and *Thermus thermophilus*, the pathway for menaquinone synthesis diverges from the one from *E. coli *with futalosine as an intermediate [35]. However the pathway is not completely elucidated so in this model, the equations of the pathway of *E. coli *were kept as in the model iIT341 GSM/GPR. As for the ubiquinone, the same three gene orthologs of the genes present in *H. pylori *have been predicted to be present in *C. jejuni*. Thiamine is essential for the growth of some strains of *Campylobacter *[36] but not for the strain NCTC11168 [32]. According to the genome annotation, the pathway for its synthesis is complete.

#### Cell wall metabolism

There is little evidence about the composition of the cell wall of *C. jejuni *in the literature, and the genome annotation suggests that the pathways are neither those of *E. coli *nor those of *H. pylori*. For instance, only 2 genes are annotated as part of the fucose biosynthesis pathway in *C. jejuni *(*cj1407c *and *cj1428c*), while 6 such genes are annotated in *H. pylori*. However in the absence of better data, the pathways for the cell wall metabolism were copied from the model iIT341 GSM/GPR. It has been shown that the fatty acid composition of *C. jejuni *changes with the environmental conditions and/or the growth rate [37,38] but the predominant fatty acid have been reported to be the saturated hexadecanoic acid, the unsaturated octodec-1l-enoic acid and to a lesser extent cyclopropane in C19 and tetradecanoic acid which increased with stress [37-40]. These are the same fatty acids that are included in the model iIT341 GSM/GPR.

#### Other

It is not clear from the genome sequence nor from the literature how *C. jejuni *assimilates sulphur, and hence we have used the assumptions used for *H. pylori *[18]. Transport reactions were mainly drawn from circumstantial evidence. Three "sink reactions" were added to the model because the pathways for the degradation of the corresponding products are unknown, and the ones used are the same as the ones introduced in *E. coli *[16] and *H. pylori *[18].

### Exploration of the predicted metabolism of *C. jejuni*

To test the model, the production of biomass from different carbon sources was simulated and the results are shown in the table in the Additional file 2: substrate utilisation. Out of the 19 substrates tested, three are not included in the model (bromosuccinate, methyl pyruvate, α-hydroxybutyrate) and for four of them, it is not clear from the literature whether they can be used as a sole carbon source or not. According to the model, glutamate, citrate, α-ketoglutarate, aspartate, asparagine, L-lactate, L and D-malate, succinate, fumarate, pyruvate and serine can be metabolised in agreement with the data of the literature. The model also allowed the metabolism of proline and L-glutamine, which did not produce significant respiration with the BIOLOG experiments [32], however they were metabolised once aspartate and serine were depleted, alongside other chemicals in the defined media [37]. It is not clear whether formate can be used as an electron donor only [41] or a carbon source as well [42,43], but cannot be used as a sole carbon source according to the microarray experiments [32] and is not predicted to be metabolised in the model. Cysteine has been shown to be metabolised [37,42] but was not predicted to be sufficient as a sole carbon source. Finally, no regulatory constraint was considered in any of the simulations, the only constraints used were the rate of consumption of the carbon source and oxygen source.

### *In silico *prediction and experimental identification of essential genes in *C. Jejuni*

The metabolic model in combination with FBA was used to predict the metabolic genes that are essential for the production of biomass in rich medium (see Materials and Methods). This generated a list of 176 predicted essential genes (see Additional file 3: predictions of essential genes).

**Figure 1:**
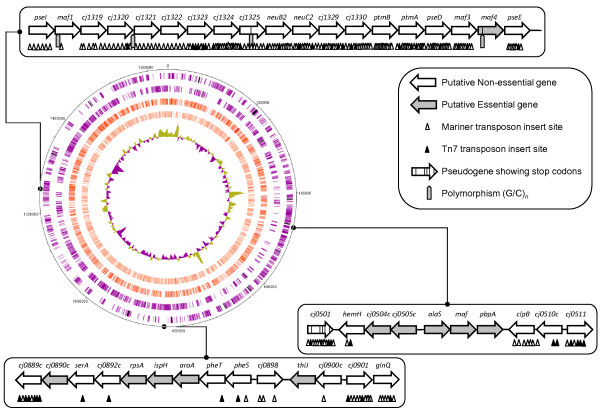
**Circular representation of the *C. jejuni *NCTC11168 genome showing positions of transposon inserts**. Outer 2 circles: open reading frames (purple) and pseudogenes (black) on the forward and reverse DNA strands. The next two circles show insert positions based on the Mariner library (dark red) and the Tn5 library (light red). The innermost circle shows %GC. Numbers on the outermost circle show the base number with respect to the start of *dnaA *(*cj0001*). Box outs show examples of the transposon mutagenesis analysis (genes are not drawn to scale). Genes are predicted essential if they lack any transposon insert (e.g. *alaS *or *aroA*) while genes with an insert are predicted to be dispensable for growth under laboratory conditions. The upper box out shows a region of the genome densely populated by transposon inserts and therefore this whole region is likely dispensable for growth under laboratory conditions. This region of the chromosome encodes functions relating to cell envelope biosynthesis and flagella modification.

Whilst the use of FBA to predict essential genes has been shown to be effective [16], it only focuses on those genes encoding proteins in the metabolic model. To make a genome-wide assessment of gene essentiality, we used random *in vitro *transposition using two transposon inserts to generate two transposon libraries in *C. jejuni *11168 (see Materials and Methods). The Mariner and Tn7 transposon libraries contained 7381 and 2169 unique inserts, respectively. Using a PCR-based genomic insertion site mapping strategy with validated primers for each annotated ORF in the *C. jejuni *genome sequence, the insertion site of each transposon was mapped as shown in Figure 1 with an accuracy of +/- 50 nt. Table 2 shows a summary of the results of the genome-wide transposon mutagenesis experiments. In total, 233 genes were identified as lacking either Tn7 or Mariner insert, and therefore potentially essential under the growth conditions used in this study. A more detailed list of insert locations is available as the Additional file 3: predictions of essential genes including the 47 directed mutants, and a summary of the proteins encoded by genes without a transposon insert, classified according to the KEGG, BioCyc, and JCVI databases, is shown in Additional file 4: genes without inserts by pathway. Such an analysis shows the predominance of essential genes associated with: aromatic amino acid metabolism, tRNA metabolism and protein synthesis, energy transduction/TCA cycle, the cell envelope, and purine and pyrimidine metabolism. There are also 27 genes (≈10% of the total in the annotated genome) with no inserts encoding hypothetical proteins of unknown function. Fifteen of these hypothetical proteins do not have homologs outside the Epsilon sub-division of the Proteobacteria and tend to be small proteins (median size, 5.6 kDa, min size, 3.6 kDa; max size, 26.4 kDa). A further subset of four genes are specific to the *jejuni *species of *Campylobacter *(*cj0416, cj0747-8*, and *cj0974*) and potentially encode small polypeptides (median size, 3.8 kDa, min size, 3.6 kDa; max size, 5.4 kDa). The *cj0416 *and *cj0747-8 *genes are subject to regulation as significant changes in transcription of these genes have been reported in microarray studies [44-47]. Thus far, no published proteomic studies in *C. jejuni *have identified these proteins, although their small size may have hampered detection in previous studies. Analysis of these small proteins with respect to the published protein-protein interaction network [48] shows that, with the exception of *cj0344 *(not present in the study), these proteins tend to be hubs, interacting with a large number of other proteins. For instance, interactions between *cj0748 *and 115 other proteins (including *cj0974*) are detected in the published network.

**Table 2:** Summary of the genome wide transposon mutagenesis for *C. jejuni*

Transposon library	Tn7	Mariner	Combined
Number of inserts measured	2251	7654	9905
Number of inserts in genes	2169	7380	9549
Percentage of inserts within genes	96.35	96.42	96.40
Number of genes with 1 or more inserts	1029	1240	1421
Number of genes with no insert	625	414	233

**Figure 2:**
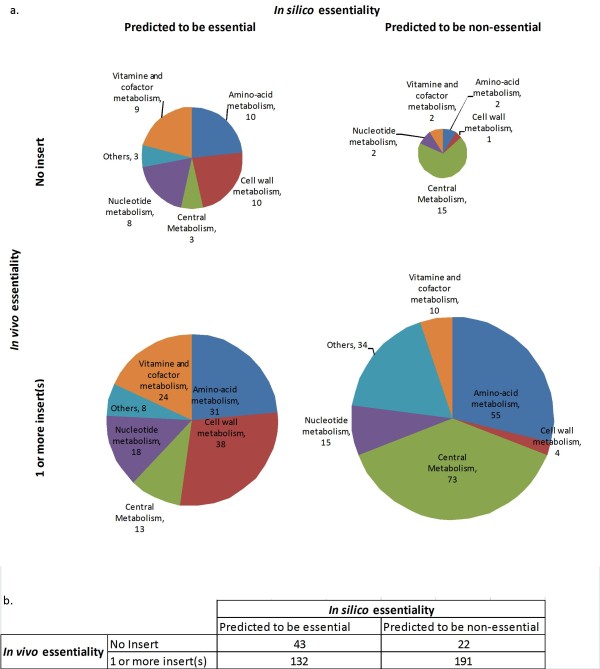
**Metabolic genes predicted to be essential or dispensable *in silico *and *in vivo *by pathway**. The genes predicted to be essential or non essential were determined by FBA (*in silico*) and from the number of inserts in a transposon mutagenesis of this study (*in vivo*) in laboratory conditions. Only the genes included in the model reconstruction for FBA are indicated. a) The pie chart shows the distribution of genes belonging to different types of pathway in each category. b) The table indicates the total numbers of genes that fall in each category.

The two lists of predicted essential genes, one resulting from FBA, and other from global *in vitro *transposition, were compared to assess both the intersection and differences. A comparison of these lists shows an intersection of 42 genes (Table 3). Assuming that the *in vitro *transposition is not biased, the model would achieve 60% accuracy (percentage of the total number of genes predicted to be essential or non essential by both methods). Figure 2 shows the distribution of the number of genes predicted to be essential or dispensable as predicted by FBA (*in silico*) and measured by the number of insert (*in vivo*) by metabolic pathway. Some of the genes predicted to be essential by FBA belonged to central metabolism, especially the gluconeogenesis pathway. Specifically, each step from phosphoenol-pyruvate to glucose-6-phosphate is predicted to be essential by FBA. However, the transposon mutagenesis only identified 3 genes involved in gluconeogenesis: *cj1403c *(glyceraldehyde 3-phosphate dehydrogenase), *cj0597 *(fructose-bisphosphate aldolase), and *cj0840c *(fructose-1,6-bisphosphatase). Nine of the 23 genes predicted to be dispensable by FBA that did not contain transposons, encode proteins associated with respiration (*cj0107, cj0936, cj1153, cj1490c, cj1566c, cj1567c, cj1569c, cj1571c *and c*j1572c*), confirming that the respiration chain of *C. jejuni*, whilst being essential for viability, is complex and not fully understood [26]. Concerning the vitamins and cofactors, predictions by FBA are uncertain because only verified transport reactions were included in the FBA model. In addition, the range of chemicals allowed in the medium for the FBA simulation was conservative because the medium composition is not well defined. This might explain the discrepancies for the biosynthesis of biotin, folic acid and pantothenate where most of the genes associated with the pathways were predicted to be essential by FBA, but were not essential according to the transposon mutagenesis. Riboflavin was assumed to be present in the medium and used by the cell [49] so it was predicted non-essential by FBA in agreement with our transposon mutagenesis data. In the transposon study of Stahl and Stintzi [20], they proposed that riboflavin biosynthesis is an essential pathway, perhaps because they used a different medium. Although ubiquinone has not been isolated from *C. jejuni *[34], the genome is predicted to encode three proteins that participate in ubiquinone biosynthesis [23]. *Cj0324 *could be attributed to the synthesis of menaquinone but *cj0546 *(UbiD) did not have any transposon inserts suggesting this gene has an unknown but important function.

**Table 3:** List of essential "metabolic genes" according to the different techniques

Technique	Essential genes
FBA only	*cj0024, cj0026c, cj0066c, cj0075c, cj0127c, cj0146c, cj0172c, cj0187c, cj0194, cj0196c, cj0197c, cj0205, cj0227, cj0237, cj0240c, cj0273, cj0274, cj0286c, cj0288c, cj0296c, cj0297c, cj0298c, cj0306c, cj0307, cj0321, cj0326, cj0332c, cj0360, cj0384c, cj0405, cj0432c, cj0433c, cj0434, cj0435, cj0437, cj0443, cj0453, cj0490, cj0541, cj0542, cj0559, cj0576, cj0580c, cj0585, cj0589, cj0638c, cj0647, cj0699c, cj0716, cj0764c, cj0766c, cj0767c, cj0795c, cj0798c, cj0806, cj0813, cj0821, cj0822, cj0847, cj0853c, cj0858c, cj0862c, cj0891c, cj0905c, cj0918c, cj0932c, cj0947c, cj0949c, cj0955c, cj0992c, cj0995c, cj1039, cj1044c, cj1046c, cj1048c, cj1067, cj1080c, cj1081c, cj1088c, cj1096c, cj1104, cj1114c, cj1133, cj1149c, cj1150c, cj1151c, cj1152c, cj1177c, cj1183c, cj1196c, cj1198, cj1202, cj1213c, cj1238, cj1243, cj1248, cj1364c, cj1398, cj1400c, cj1401c, cj1402c, cj1404, cj1407c, cj1424c, cj1428c, cj1476c, cj1498c, cj1515c, cj1529c, cj1530, cj1605c, cj1634c, cj1641, cj1645, cj1672c, cj1685c*

FBA & transposon mutagenesis of this study	*cj0027, cj0116, cj0117, cj0324, cj0387, cj0394c, cj0442, cj0514, cj0516, cj0581, cj0597, cj0639c, cj0641, cj0686, cj0840c, cj0894c, cj0927, cj1008c, cj1045c, cj1054c, cj1058c, cj1082c, cj1131c, cj1250, cj1274c, cj1288c, cj1346c, cj1347c, cj1366c, cj1403c, cj1443c, cj1536c, cj1607, cj1610, cj1652c*

FBA & transposon mutagenesis of Stahl and Stintzi	*cj0132, cj0147c, cj0308c, cj0356c, cj0451, cj0503c, cj0722c, cj0845c, cj0861c, cj0925, cj1201, cj1239, cj1290c, cj1291c, cj1531, cj1535c*

FBA & the 2 transposon mutagenesis methods	*cj0231c, cj0545, cj0707, cj0810, cj0855, cj0895c, cj1644, cj1676*

**Figure 3:**
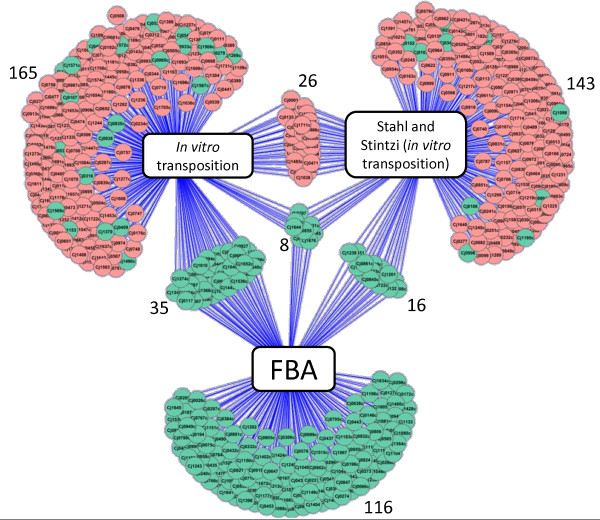
**Genes predicted to be essential by FBA and by two independent transposon mutageneses**. The essential genes determined by FBA include "metabolic genes" only, shown in green. The "non-metabolic genes" are indicated in red. The genes predicted to be essential by transposon mutagenesis were those with no insert in the transposon mutagenesis in this study and in the study of Stahl and Stintzi [20].

The "metabolic genes" refer to the genes that were included in the FBA model. This table gives the list of the genes shown in Figure 3. The genes predicted to be essential by transposon mutagenesis were those with no insert in the transposon mutagenesis in this study and in the study of Stahl and Stintzi [20].

One important caveat of the essential gene predictions using FBA is that some pathways were copied from *H. pylori*. In addition many genes annotated to be part of the cell wall were not taken into account in the model. However many genes involved in fatty acid metabolism and all the genes involved in fatty acid elongation are predicted to be essential by FBA: *cj0328c *(FabH), *cj1303 *(FabH2), *cj0442 *(FabF), *cj0435 *(FabG), *cj0273 *(FabZ), and *cj1400c *(FabI). FabD (*cj0116*) and FabF are also predicted essential from the transposon study. Fatty acid biosynthesis in *Campylobacter *is likely to contribute to the biosynthesis of the cell envelope, so it is perhaps not surprising that this is a key pathway.

### Comparison of the essential gene predictions with a published study

**Figure 4:**
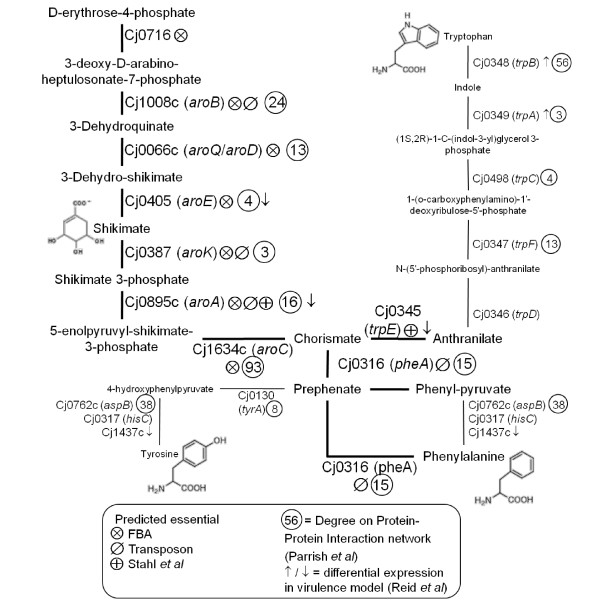
**The shikimate pathway**. The pathway shows the biosynthesis of aromatic amino acids, via shikimate, as suggested by the genome annotation. Each step is annotated to show the following: the enzyme ('Cj' number and gene symbol), the prediction of essentiality based on FBA and transposon mutagenesis, the number of interactions in the PPI network, and the direction of expression from microarray data from a piglet model of infection. Predicted essential steps are shown in bold.

The predictions made in this study were further compared to the published transposon mutagenesis study of Stahl and Stintzi [20]. The greatest overlap is between FBA and our transposon list (35 genes) as illustrated in Figure 3 and listed in Table 3. There are only 26 genes that overlap between our transposon study and the published study of Stahl and Stintzi [20]. Eight genes are common to all three lists although they encode proteins from a number of disparate pathways (*cj0231c *(NrdF), *cj0545 *(HemC), *cj0707 *(KdtA), *cj0810 *(NadE), *cj0855 *(FolD), *cj0895c *(AroA), *cj1644 *(IspA), *cj1676 *(MurB)). An analysis of the intersection genes, with respect to the KEGG, JCVI, and BioCyc databases, shows clusters of genes associated with: purine and pyrimidine metabolism, the cell envelope, cofactor biosynthesis (menaquinone/ubiquinone, folic acid, biotin, heme), and a cluster of five hypothetical proteins with no known function (*cj0364, cj0703, cj0711, cj0939c*, and *cj1712*). Following further analysis of these protein sequences using BLAST and the Pfam database, *cj1712 *is revealed to be a putative PunB/DeoD homolog (purine nucleoside phosphorylase) involved in purine and pyrimidine metabolism as shown by the amino acid sequence containing a Phosphorylase superfamily domain (E = 1.3e-07) and showing between 40-80% identity to other proteins annotated as PunB/purine nucleoside phosphorylases in the NCBI microbial database. Three genes from the aromatic amino acid biosynthetic pathway (specifically, the shikimate pathway) are also present in the intersection list. Further analysis of this pathway, shows that nine genes encoding proteins from this pathway are predicted to be essential by one or more of the techniques used in this study (Figure 4). Furthermore, analysis of these proteins with respect to the published protein-protein interaction network [48] reveals that many proteins from this pathway have a high number of interactions with other proteins and may therefore be classified as 'hubs' in the metabolic network (Figure 4). Interestingly, a number of these genes are also subject to transcriptional regulation in a virulence model [46]. Taken together, these data point to biosynthesis of aromatic amino acids as a potential metabolic weak-point in *Campylobacter*, which is also relevant during infection.

## Discussion

In this study we have combined bioinformatic approaches to construct and validate a genome-wide model of metabolism of *Campylobacter jejuni*, the first such model of this important food-borne pathogen. Flux Balance Analysis has been used to predict those proteins that, if removed from the model, result in loss of biomass production. To complement this *in silico *predictive approach, we have used random transposon mutagensis coupled to gene-specific PCR to identify those genes that contain one or more transposon insert (dispensable genes for growth under laboratory conditions) and those genes that do not contain a transposon insert, the putative essential genes.

### *In silico *determination of essential genes

Although the reconstruction of the metabolic network of *C. jejuni *is based on limited biochemical data, it was possible to formulate a hypothesis on the metabolism of this pathogen. The reconstruction pointed out the main areas of uncertainty: the cell wall metabolism and nucleotide pathways. It was also found that the pathway for sulphur assimilation is not obvious from the genome annotation.

A malate synthase activity was an assumption in our model, and this activity has been demonstrated in *H. pylori *[29]. Based on the annotation, the genome does not encode a malate synthase, and extensive BLAST searching using both the malate synthase A (*aceB*) and malate synthase G (*glcB*) sequences did not reveal any match in the *H. pylori *or *C. jejuni *genomes or any genome from the epsilon sub-division of Proteobacteria. A new class of malate synthase enzymes may be present in *H. pylori *and possibly in this clade of life, that does not have sequence homology to known characterised malate synthase enzymes from other bacteria, although this requires further biochemical evidence. The reconstruction of the model was mainly based on conventional genome annotation employing BLAST searches. More sophisticated annotation methods have been proposed to address functional divergence amongst proteins that share sequence similarity [50,51]. For instance, we compared the EC numbers obtained with the EFICAz [52] and PRIAM [53] tools to the EC numbers of the reactions linked to a unique gene in our model (287 reactions) and found discrepancies for 30 and 27 reactions respectively. Based on the original genome annotation, some enzymes in our model could catalyse diverse reactions, while the more sophisticated annotation tools suggested more metabolic specificity. An example is *cj0324*, originally annotated as a ubiquinone/menaquinone methyltransferase (EC 2.1.1.-) [23], the PRIAM tool [53] suggests specifically demethylmenaquinone methyltransferase activity (EC 2.1.1.163), which is more likely as menaquinone and a methyl substituted menaquinone have been isolated in *C. jejuni *rather than ubiquinone [34]. We also checked our metabolic model against the 'expert community' subsystem annotation presented in The SEED [54], which returned discrepancies for 20 of the reactions considered above. For some genes, the precise annotation depends on the method used: returning to our glycolate to glyoxylate interconversion hypothesis, Cj1213c is a putative glycolate oxidase subunit D (EC 1.1.3.15) [23,55], or an alkylglycerone-phosphate synthase (EC 2.5.1.26) [52], or a D-lactate dehydrogenase (EC 1.1.2.4) [53] making the degradation of glycolate into glyoxylate an uncertain assumption.

An inherent limitation of the FBA method is the suitability of the objective function [56]. For instance, by optimising the biomass, FBA does not take into account the microaerophilic and capnophilic properties of *C. jejuni*. These may indeed constitute additional constraints like a maximum concentration of oxygen-sensitive enzymes neglected in these simulations except as modelled by the limiting uptake rate of oxygen. Alternatively, trade-off functions may be more appropriate objective function than the optimisation of the biomass with these kind of micro-organisms [57].

The FBA method has the potential of being condition specific to determine the essential genes. In this study, they were determined in laboratory conditions. However as more data become available on the conditions in the chicken gut, the model has the potential of being used in situations relevant to the food industry.

### Transposon methods

Flux balance analysis methods have a good track record of predicting essential genes [16], however, they only focus on metabolism-related genes. In this study, the FBA model only contained reactions linked to 388 genes, 24% of the total genome. *In vitro *transposition has the advantage of targeting the whole genome. We describe the construction of two transposon mutant libraries in *C. jejuni *NCTC11168 and the mapping of a total of 9550 inserts in the genome, this represents a coverage of 5.94× using the method of Stahl and Stintzi [20]. Data from the combined transposon mutagenesis libraries predicted 233 essential genes (14% of the genome total). While FBA only uses a subset of genes from the genome, the number of predicted essential genes was similar at 175 (11% of the genome). These numbers are similar to the published *C. jejuni *study (194 genes, 12% of the genome) although the overlap between the published study and the data presented in this study is comparatively small (only 8 genes predicted by all three methods). Compared to other published essential gene prediction studies, the number of predicted essential genes is between the lower quartile and median with respect to total number of essential genes and percentage of the genome predicted to be essential. However, reviewing all published microbial essential gene predictions, we noted there was no correlation between number of predicted essential genes and genome size (data not shown). Clearly the relationship between genome size, complexity of niche, and indispensable genes is complex, plus a number of caveats should always be considered when interpreting this sort of data: any gene containing at least one insert can be said to be non-essential under the growth conditions described. However, the inverse logic is not true. The absence of an insert in a gene does not necessarily mean that the gene is required for growth and hence essential. Transposon insertion may not have occurred for a variety of reasons: chance, sequence bias of the transposase or transposon depletion during the reaction. Although no detailed studies of the sequence preference of either transposases used here have been carried out and it is generally assumed they are essentially random, we used two different transposases in an attempt to minimise any effect of sequence bias. An over-representation of small hypothetical proteins (and accordingly, small genes lacking an insert) may have resulted from the random nature of the transposon insertion: i.e. the smaller the gene, the smaller the chance of transposon insertion; however, no gene size bias was observed when comparing genes with insert with genes encompassing the entire genome.

The genome of *C. jejuni *NCTC 11168 totals 1,641,481 bases and thus our libraries represent insertions in only ~0.006% of the possible positions. It is possible that the number of inserts identified is an under representation of the actual total since inserts close together would generate very similar sized PCR products that may fail to be discriminated on the agarose gels. Additionally, the abundance of individual mutants in the isolated pooled genomic DNA may also affect whether a band is visible. Since the genomic DNA used was isolated from pooled colonies, it is possible that any mutant resulting in reduced growth and hence colony size would be under represented in the pooled material and as a consequence, would not be detected. The whole genome *in vitro *transposition presented in this study should be seen as a high-throughput method, as opposed to a high precision method. A number of caveats should not be ignored: the library unlikely represents all possible insertion points and some regions may be naturally more resistant to accepting an insert. Detection is constrained by the primer library, which in this case was optimized for microarray probe generation. The PCR and agarose gel-based approach also suffers from more common technical drawbacks such as smaller PCR products are more favourably amplified that longer ones and accuracy of sizing gel fragments is not infallible. Additionally, polar effects due to operon structure, may result in the null recovery of some mutants, if the transcription and translation of upstream genes is perturbed by a transposon insert, as was shown for the *C. jejuni *fur gene which in itself is not essential [58]

### Functional vs. topological determination of essential genes

Another source of information which covers a high percentage of the genome is the Protein-Protein Interaction (PPI) network of *C. jejuni*, obtained by yeast two-hybrid methods which covers 80% of the proteome [48]. Each protein is a node and if they interact, they are linked by an edge. Essential genes have been linked to the topological properties of the PPI network, as it has been shown that essential genes are more likely to be hubs of the PPI network than by chance [59,60]. We have investigated whether there is a correlation between the degrees of the nodes the PPI network of *C. jejuni *and essential genes determined using FBA and transposon mutagenesis. No correlation was observed, contrarily to what was predicted by Parrish *et al. *[48]. They based their analysis on putative essential proteins which were orthologs of *Escherichia coli *and *Bacillus subtillis *essential proteins. However, it has been shown that these bacteria do not share many essential genes, especially *B. subtillis *[20]. More recent analyses of binary PPI networks suggest that the relationship between hubs and essential proteins is more complex, with hubs being correlated to genetic pleiotrophy; that is hubs are proteins that have many phenotypes when the gene encoding that protein is deleted [61]. The interpretation of PPI networks remains ambiguous and models to explain the universal structure of PPI networks have been proposed to be related to evolutionary principles such as duplication and mutation of a few ancestors [62] or to the potential of proteins to bind together because of their physical properties, such as binding affinity and folding [63].

### The shikimate pathway

Whilst the genome-wide comparison of gene essentiality with PPI hubs has not been fruitful in this study, the shikimate pathway in particular exhibits a large number of proteins with high degrees (see Figure 4). Since the interpretation of physical PPI network remains ambiguous, the high degrees of this pathway could be interpreted in multiple ways. Nonetheless, the combination of essential gene prediction methods has drawn focus to this particular pathway as a potential target for intervention, which should be investigated further using conventional genetic tools. The shikimate pathway has been the subject of antimicrobial research in previous studies [21,64,65]. As reported by other groups, the shikimate pathway is present in bacteria, plants, and fungi, but absent in humans, making it the target for novel antimicrobials and herbicides [21]. More specifically, Zucko *et al. *show that a complete shikimate pathway is present in 76% of 442 bacterial genomes studied, although largely incomplete in Archaea [66]. Two *E. coli *studies also identify essential genes from this pathway: *aroH*, *aroK *were predicted essential by Gerdes *et al. *[67] and *aroB*, *aroD*, *aroE*, *aroC*, and *pheA *plus the entire *trpABCDE *operon were predicted essential by Joyce *et al. *[68]. The *aroD *gene is also predicted to be essential in the refined *H. pylori *metabolic model [18].

It is noteworthy that without any prior expectations of pathway targets, the methods presented in this work point towards a known target pathway for novel antimicrobial interventions. However, the ultimate validation of our approach requires further laboratory investigation that is beyond the scope of this paper.

## Conclusions

We have presented the first curated metabolic model of the important pathogen *Campylobacter jejuni *and discussed insights into the organism's metabolism. Flux Balance Analysis used in combination with a transposon mutagenesis library has been used to make predictions about essential genes, and these predictions have been further informed with reference to other published studies, such as the PPI dataset. This analysis has provided the basis for further laboratory investigations and suggests a re-evaluation of a previously scrutinized pathway, which may turn out to be the Achilles heel of this food-borne pathogen.

## Methods

### Reconstruction of the metabolic network for FBA

The reconstruction of the metabolic network is based on the genome sequence of *C. jejuni *NCTC 11168 [23] and a recently curated and updated annotation [22]. Where *C. jejuni *had *H. pylori *orthologs (they share about 2/3 of their genome), the reactions were taken from the *H. pylori *model iIT341 GSM/GPR [18] with the same assumptions. The reactions were also checked against on-line databases [69,70] and also the literature on *C. jejuni*. In particular, the reactions for the central metabolism and respiration were drawn from a recent review [24]. The conventions for the names of chemicals and reactions were kept as close as possible to the *H. pylori *model ilT341 GSM/GPR. The reactions added to the model were elementary and charge balanced based on a neutral intracellular pH. Where possible, the reactions were associated to genes that encode the proteins which catalyse them, with Boolean relationships. This means that for reactions catalysed by isozymes or different proteins, the "and", "or" Boolean operations between the genes was used [71].

### Validation test for the model

To check that the model allowed the bacteria to metabolise the expected substrates, it was tested against BIOLOG microplates where the respiration of *C. jejuni *fed on different carbon source was measured [32], and, other literature data [37,42,43,72]. Due to the scarcity of data in the literature, the model could only be tested with oxygen for respiration.

### Prediction of essential genes by FBA

FBA consists of the computation of the possible fluxes, ν, going through the reactions of the metabolic network at steady-state. The system of equations is defined by the stoichiometric matrix, *S*, containing the stoichiometric coefficients of the metabolic reactions, with *m *being the number of metabolites, and *n *the number of fluxes [15]. At steady-state (i.e. during balanced growth when the biomass composition is assumed to be constant), *S.ν = *0. There are more fluxes than metabolites (*n *>*m*), so the system is underdetermined. The fluxes are bound by thermodynamic feasibility so the space of solutions is a convex space [15]. It has been shown with some organisms, notably with *E. coli *[16], that the flux through the biomass is optimised for the uptake of nutrients during balanced growth, so the biomass equation can be used as an objective function to reduce the space of solutions. With less well characterised micro-organisms like *C. jejuni*, the biomass composition is not known quantitatively. The biomass composition was assumed to be the same as for *H. pylori *except for (a) vitamin B6, which was added to the equation as the genome is predicted to encode the entire biosynthetic pathway bar one gene and (b) thiamine, for which the active form was assumed to be thiamine diphosphate rather than thiamine.

Exploration of the space of solution: Even after appointing an objective function, there may be more than one solution to the optimisation problem. These solutions are referred to as silent phenotypes as the growth rate is the same but the internal organisation of the fluxes is different [73]. Due to the design of the algorithm, the solution returned by simplex linear programming generally minimizes the number of fluxes.

To estimate whether a gene is essential or not, the ratio, *Gr*, of the biomass flux when the gene is absent to the biomass flux when the gene is present was calculated in a given environment [74]. A gene was considered essential if *Gr *<= 10^-9 ^(arbitrary value).

Medium composition: Minimal medium was used to validate the model. Its substrates were derived from the BIOLOG medium experiments http://www.biolog.com. The experiments to screen for essential genes were carried out in Brucella medium which is a rich medium containing pancreatic digest of casein, peptic digest of animal tissues, dextrose, yeast extract, sodium chloride and sodium bisulfite [75]. Since the composition of this medium is unknown, the medium was assumed to be similar to yeast extract [76], the composition used for simulations is indicated in the table in the Additional file 5: medium composition. The chemicals are allowed to enter or leave the system through exchange reactions. The exchange fluxes of the carbon source were fixed to a maximum of 20 mmol/g dry weight of biomass/h, which is close to a maximum uptake rate for *E. coli *[77] and 5 mmol/g dry weight of biomass/h for oxygen, which is about a fourth of the maximum fluxes measured in air for *E. coli *as *C. jejuni *is microearophilic. The other nutrients present in the medium were assumed to be non-limiting with an arbitrary uptake higher boundary of 1,000 mmol/g dry weight of biomass/h.

All the calculations related to FBA were carried out with the COBRA Toolbox [74] in Matlab (version R2010b, Mathworks, Inc.) equipped with the glpk solver for linear programming [78].

### Transposon mutagenesis

Two *in vitro *transposition libraries were constructed using mariner transposase, essentially as described by Gaskin and van Vliet [79] and Tn7 transposase from New England Biolabs with *C. jejuni *NCTC11168 genomic DNA. These were introduced into *C. jejuni *NCTC 11168 cells by natural transformation [79] and plated onto Blood Agar Base no.2 (Oxoid) plates supplemented with 5% v/v defribinated horse blood and kanamycin 50 μg/ml. After ~48 hours incubation at 42°C under microaerophilic conditions (5% oxygen, 10% carbon dioxide, 85% nitrogen) colonies were pooled and genomic DNA extracted using QIAgen genomic-tips (QIAgen).

### Mapping of transposon insertions

Genomic DNA from the pooled colonies was used as template in polymerase chain reactions using a transposon specific primer and individual gene specific primers. Briefly, 50 μl reactions were set up using 100 ng genomic DNA, 10-50 pmol of each primer and 25 μl of HotStarTaq mix (QIAgen). Cycling conditions were 95°C for 15 min, followed by 30 cycles of 95°C 30 sec, 50°C 30 sec, 72°C 90 sec and a final 72°C 15 min extension step. Aliquots from each reaction were run on 0.8% agarose gels, which were stained with ethidium bromide. Gel images were captured using a GeneDoc system (Anachem). The sizes of observed bands were calculated using Labimage (Kapelan Bio-Imaging GmbH) and this data was inputted into Excel (Microsoft). Based on the transposon study, 47 insertional inactivation mutants were created using conventional methods to validate the Tn7/Mariner findings.

## Authors' contributions

AM reconstructed the metabolic network, carried out the FBA simulations and PPI network analysis. MR participated in the reconstruction of the metabolic network and analysed the results. DHJG carried out the transposon mutagenesis. AM, MR and DHJG drafted the manuscript. JB and AHMV conceived the study and critically revised the manuscript. All authors read and approve the final manuscript.

## Supplementary Material

Additional file 1 **Tables with the reactions included in the model, the metabolites and the references use to reconstruct the model**. In the reaction table, the EC numbers of the reactions are indicated as well as the *H. pylori *orthologs as appropriate. The reactions are associated to genes with Boolean rules. The reactions were categorised in pathways similarly to the model of *H. pylori*. Click here for file

Additional file 2 **This table shows the substrates that were predicted to be metabolised according to the proposed model and compared to experimental data from the literature**. Click here for file

Additional file 3 **This table shows whether genes were predicted to be dispensable by different methods: FBA predictions, number of inserts of the transposon mutagenesis for the two transposon libraries of this study and whether inserts were present in the transposon mutagenesis of an independent study **[20]. The degrees of the protein in the PPI network and known mutants are also indicated. Click here for file

Additional file 4 **This table is a list of the proteins encoded by genes without a transposon insert as determined in this study (putative essential genes), classified according to the KEGG, BioCyc, and JCVI databases**. Click here for file

Additional file 5 **Table of substrates allowed in the system to determine essential genes *in silico***. Click here for file
